# Changes in quality of life, cognition and functional status following catheter ablation of atrial fibrillation

**DOI:** 10.1136/heartjnl-2020-316612

**Published:** 2020-10-12

**Authors:** Jonathan P Piccini, Derick M Todd, Tyler Massaro, Aimee Lougee, Karl Georg Haeusler, Benjamin Blank, Joseph Paul de Bono, David J Callans, Arif Elvan, Thomas Fetsch, Isabelle Van Gelder, Philip Gentlesk, Massimo Grimaldi, Jim Hansen, Gerhard Hindricks, Hussein Al-Khalidi, Lluis Mont, Jens Cosedis Nielsen, Georg Noelker, Tom De Potter, Daniel Scherr, Ulrich Schotten, Sakis Themistoclakis, Johan Vijgen, Luigi Di Biase, Paulus Kirchhof

**Affiliations:** 1 Division of Cardiology, Duke University Medical Center & Duke Clinical Research Institute, Durham, North Carolina, USA; 2 Cardiology, Liverpool Heart and Chest Hospital, Liverpool, UK; 3 Biostatistics, Duke University Medical Center, Durham, North Carolina, USA; 4 Biostatistics, Duke Clinical Research Institute, Durham, North Carolina, USA; 5 Department of Neurology, Universitätsklinikum Würzburg, Wurzburg, Germany; 6 Atrial Fibrillation NETwork association (AFNET), Munster, Germany; 7 Institute of Cardiovascular Sciences, University Hospital Birmingham, Birmingham, West Midlands, UK; 8 Medicine, University of Pennsylvania School of Medicine, Philadelphia, Pennsylvania, USA; 9 Cardiology, Isala Klinieken, Zwolle, The Netherlands; 10 Cardiology, The Clinical Research Institute, Muncher, Germany; 11 Cardiology, University Medical Centre Groningen Thorax Centre, Groningen, The Netherlands; 12 Cardiology, Sentara Norfolk General Hospital, Norfolk, Virginia, USA; 13 Ente Ecclesiastico Ospedale Generale Regionale Francesco Miulli, Acquaviva delle Fonti, Puglia, Italy; 14 Department of Cardiology, Gentofte Hospital, Gentofte, Hovedstaden, Denmark; 15 Cardiology, University of Leipzig, Leipzig, Germany; 16 Duke Clinical Research Institute, Durham, North Carolina, USA; 17 Cardiovascular Institute, University of Barcelona, Barcelona, Spain; 18 Cardiology B, Aarhus University Hospital, Aarhus N, Denmark; 19 Department of Cardiology, Heart and Diabetes Center North Rhine-Westphalia, Ruhr University Bochum, Bad Oeynhausen, Germany; 20 Cardiology, Onze Lieve Vrouw Ziekenhuis, Aalst, Belgium; 21 Cardiology, Medical University of Graz, Graz, Austria; 22 Department of Cardiology, Cardiovascular Research Institute Maastricht (CARIM), Maastricht University Medical Center, Maastricht, Limburg, Netherlands; 23 Physiology, Maastricht University Medical Centre, Maastricht, The Netherlands; 24 Cardiology, Ospedale Dell'Angelo, Mestre-Venice, Italy; 25 Heart Center, Jessa Hospital, Hasselt, Belgium; 26 Cardiology, Montefiore Medical Center, Bronx, New York, USA; 27 SWBH NHS Trust, Birmingham, UK

**Keywords:** atrial arrhythmia ablation procedures, atrial fibrillation, quality and outcomes of care

## Abstract

**Objective:**

To investigate changes in quality of life (QoL), cognition and functional status according to arrhythmia recurrence after atrial fibrillation (AF) ablation.

**Methods:**

We compared QoL, cognition and functional status in patients with recurrent atrial tachycardia (AT)/AF versus those without recurrent AT/AF in the AXAFA–AFNET 5 clinical trial. We also sought to identify factors associated with improvement in QoL and functional status following AF ablation by overall change scores with and without analysis of covariance (ANCOVA).

**Results:**

Among 518 patients who underwent AF ablation, 154 (29.7%) experienced recurrent AT/AF at 3 months. Patients with recurrent AT/AF had higher mean CHA_2_DS_2_-VASc scores (2.8 vs 2.3, p<0.001) and more persistent forms of AF (51 vs 39%, p=0.012). Median changes in the SF-12 physical (3 (25th, 75th: −1, 8) vs 1 (−5, 8), p=0.026) and mental scores (2 (−3, 9) vs 0 (−4, 5), p=0.004), EQ-5D (0 (0,2) vs 0 (−0.1, 0.1), p=0.027) and Karnofsky functional status scores (10 (0, 10) vs 0 (0, 10), p=0.001) were more favourable in patients without recurrent AT/AF. In the overall cohort, the proportion with at least mild cognitive impairment (Montreal Cognitive Assessment <26) declined from 30.3% (n=157) at baseline to 21.8% (n=113) at follow-up. ANCOVA identified greater improvement in Karnofsky functional status (p<0.001) but not SF-12 physical (p=0.238) or mental scores (p=0.065) in those without recurrent AT/AF compared with patients with recurrent AT/AF.

**Conclusions:**

Patients without recurrent AT/AF appear to experience greater improvement in functional status but similar QoL as those with recurrent AT/AF after AF ablation.

## Introduction

Catheter ablation is increasingly employed treatment option for rhythm control in patients with atrial fibrillation (AF).^1^ While some clinical trials have shown that catheter ablation can improve cardiovascular outcomes in certain patient groups, the primary indication for catheter ablation in current practice is to improve symptoms and quality of life (QoL).^1–4^ Interestingly, improvements in symptom burden and QoL have been observed even in patients with recurrent AF.^5^

Despite evidence that catheter ablation is more effective than antiarrhythmic drug therapy for the treatment of recurrent AF, catheter ablation is associated with infrequent but measurable periprocedural risks, including stroke. Moreover, high-resolution diffusion weighted brain MRI identifies acute brain lesions without neurological symptoms in 10%–40% of patients undergoing catheter ablation.^6 7^ MRI-detected acute brain lesions may contribute to cognitive decline in patients who are treated with catheter ablation. These potential adverse effects of catheter ablation are concerning, especially in view of the mainly symptomatic benefits for patients.^8 9^

In order to examine the impact of the results of catheter ablation on symptoms, QoL, and cognition, we compared these important outcomes in patients with and without recurrent AF in the AXAFA clinical trial.^10^

## Methods

The rationale and design of AXAFA–AFNET 5 (*A*nticoagulation using the direct factor *Xa* inhibitor apixaban during *A*trial *F*ibrillation catheter *A*blation: Comparison to vitamin K antagonist therapy) have been described previously.^10^ In brief, AXAFA-AFNET 5 was an investigator-initiated, prospective, parallel-group, randomised, open, blinded outcome assessment study comparing continuous apixaban therapy to vitamin K antagonist therapy during ablation. AXAFA-AFNET 5 was conducted in Europe and North America. The trial sponsor was AFNET, Münster, Germany (www.kompetenznetz-vorhofflimmern.de). AXAFA–AFNET 5 was designed by the steering committee in cooperation with AFNET and conducted in accordance with the declaration of Helsinki and the International Conference on Harmonization Good Clinical Practice Guidelines. The protocol was approved by ethical review boards at all institutions. The Clinical Research Institute (CRI, Munich, Germany) executed the study in cooperation with the steering committee and the sponsor. Data collection and entry was performed using the MARVIN eCRF system.^10^ An independent steering committee and an independent data and safety monitoring board guided the trial. Patients were not involved in the design of the trial. All adverse events were adjudicated by an independent endpoint review committee blind to study group and INR values. The Duke Clinical Research Institute served as the statistical core and performed the statistical analyses for the trial. The authors vouch for the accuracy and completeness of the data and for the fidelity of the trial to the protocol. This manuscript was written by the authors.

### Study population

AXAFA–AFNET 5 enrolled patients scheduled for a de novo/first AF ablation with at least one established stroke risk factor (age >65 years, heart failure, hypertension, diabetes or prior stroke). For the purpose of this analysis, the study population included all patients from the AXAFA trial population who were randomised, underwent catheter ablation and had available baseline and follow-up QoL data.

### Measures and outcomes

Several QoL and cognitive function measures were prospectively collected in AXAFA at baseline and 3-month follow-up, including EQ-5D, SF-12, modified European Heart Rhythm Association (mEHRA) classification, Karnofsky performance status, and Montreal Cognitive Assessment (MoCA) Test scores.

The EQ-5D was developed by the EuroQol group and is a short, standardised measure of generic health. The EQ-5D assesses five dimensions across three response levels: mobility, self-care, usual activities, pain/discomfort and anxiety/depression. For the purpose of this analysis, the summary index was used in which the score ranges from 0 to 1, where higher scores reflect better health status.

The SF-12 is an abbreviated 12-item generic health related QoL measure derived from the Short Form 36. The measure evaluates physical functioning, limitations due to physical health problems, bodily pain, energy/fatigue, social functioning, limitations due to emotional problems, and psychological distress and well being. The SF-12 is reported as the physical component summary (PCS) and mental component summary (MCS). Higher scores indicate better health status. Samsa and colleagues defined the minimal clinically important difference (MCID) for the PCS as 3 in a cardiovascular (CV) population^11^ and Clement and colleagues^12^ have calculated the MCID as 2.7 (in a non-CV population). MCID values for the MCS are more variable and have not been well-quantified in patients with cardiovascular disease. In patients with joint disease, the reported absolute MCID values range from 1.4 to 4.5.^13^

The mEHRA classification assesses symptoms and functional limitation due to AF. The score is ordinal where class 1 represents no symptoms, class 2a represents mild symptoms (not troublesome to patient), class 2b represents moderate symptoms (troublesome to patient), class 3 represents severe symptoms (impacts normal daily activity) and class 4 represents disabling symptoms (normal daily activity is discontinued).^14^

The Karnofsky performance score is an instrument used to evaluate functional capacity. Originally developed in oncology, the score has been used in assessments of cardiovascular interventions.^15^ The Karnofsky score ranges from 100 to 0, where 100 is ‘perfect’ health and 0 is death.

The MoCA evaluates global cognition by assessing short term memory, visuospatial abilities, executive function, attention, concentration and working memory, language and orientation to time and place. The MoCA has 30 test items and can be administered in approximately 10 min. It is scored between 0 and 30 with higher values indicating better cognitive function. A score of 26 or higher indicates normal cognitive function.^16^

Changes in quality-of-life and cognitive function compared with baseline were prespecified secondary outcomes in AXAFA. As per study design, we also assessed freedom from atrial tachycardia (AT) or AF postablation (referred to as AF hereafter) after a 3-month blanking period as per consensus definition.

### Statistical analysis

Changes in QoL, cognitive function and functional status were assessed at 3 months compared with baseline using the EQ-5D and SF-12 questionnaires, MoCA, and Karnofsky scale. Changes in QoL and cognitive function were evaluated separately by analysis of covariance (ANCOVA) models (SF-12 physical component scores, SF-12 mental component scores, Karnofsky scores and MoCA). The ANCOVA model included the recurrent AT/AF status as an indicator variable and adjustment for the baseline quality-of-life values. Multiple linear regression was conducted on changes in 3-month SF-12 scores (MCS and PCS) using clinically important baseline measures. These measures included age (years); sex; weight (kg); body mass index (kg/m^2^); systolic and diastolic blood pressure (mm Hg); Cockcroft-Gault estimated creatinine clearance (mg/dL); New York Heart Association functional classification; prior history of diabetes, stroke or transient ischaemic attack, vascular disease (coronary, peripheral, or carotid), mitral valve disease, aortic valve disease or chronic obstructive pulmonary disease; prior major bleeding; type of AF (paroxysmal versus persistent/long-standing persistent AF); modified EHRA (I, IIa, IIb, III, IV); MoCA; and rhythm at start of ablation (sinus rhythm, AF, atrial flutter, pacing, or other). A sensitivity analysis was conducting using multiple logistic regression assessing the difference in the proportion of patients showing a 2.5 point or more improvement in their MCS and PCS scores. Descriptive statistics for continuous and categorical variables were summarised as means (SDs), median (25th, 75th percentiles) and counts (percentages), respectively. Unadjusted statistical comparisons between continuous variables were performed using the Wilcoxon rank-sum test or two-sample t-test depending on normality; comparisons between nominal variables were performed using the Pearson’s χ² test or Fisher’s exact test, depending on expected cell sizes (n<5). All analyses were two-sided and tested at the nominal 0.05 significance level, and no adjustment was made for multiple testing.

Statistical analyses were performed with SAS V.9.4 (SAS Institute, Cary, North Carolina, USA).

## Results

### Baseline characteristics

Among 518 patients undergoing ablation with available QoL data (82% of all patients undergoing AF ablation), 154 (29.7%) experienced recurrent AT/AF at the end of follow-up (3 months). Patients with recurrent AT/AF had higher CHA_2_DS_2_-VASc scores (3 (25th, 75th: 2, 3) vs 2 (1,3), p<0.001), more frequently had heart failure (37.7% vs 29.1%, p=0.04), prior stroke or transient ischaemic attack (11.7% vs 6.0%, p=0.028), coronary artery disease (17.5% vs 10.7%, p=0.033) and prior major bleeding (5.2% vs 0.8%, p=0.004) compared with patients who had no recurrent AT/AF (table 1). In terms of medical therapy, patients with recurrent AT/AF were less likely to be on flecainide (13.6% vs 22.3%, p=0.024). Patients with recurrent AT/AF were less likely to have paroxysmal AF (49.4% vs 61.3%, p=0.012) or to be in sinus rhythm at the time of ablation (56.5% vs 74.5%, p<0.001).

**Table 1:** Baseline characteristics in those with and without recurrent AT/AF*

	All patients	Recurrent AT/AF	No recurrent AT/AF	P value
	n=518	n=154	n=364	
Age, median (q1, q3)	64 (58 to 70),	65 (60 to 70),	64 (57 to 70),	0.076
Female	174 (33.6%)	61 (39.6%)	113 (31.0%)	0.059
Weight, median (q1, q3)	87 (76 to 98)	88 (77 to 101)	86 (76 to 97)	0.231
BMI, median (q1, q3)	28 (25 to 31)	29 (26 to 32)	28 (25 to 31)	0.184
CHA_2_DS_2_VASc score, median (q1, q3)	2 (2 to 3)	3 (2 to 3)	2 (1 to 3)	<0.001
Hypertension, n (%)	468 (90.3%)	136 (88.3%)	332 (91.2%)	0.307
Systolic blood pressure, median (q1, q3)	140 (125 to 151)	139 (125 to 151)	140 (125 to 150)	0.990
Diastolic blood pressure, median (q1, q3)	83 (76 to 90)	85 (76 to 92)	81 (75 to 90)	0.145
COPD, n (%)	33 (6.4%)	8 (5.2%)	25 (6.9%)	0.476
Heart failure	164 (31.7%)	58 (37.7%)	106 (29.1%)	0.040
NYHA I	48 (9.3%)	18 (11.7%)	30 (8.2%)	
NYHA II	98 (18.9%)	30 (19.5%)	68 (18.7%)	
NYHA III	18 (3.5%)	10 (6.5%)	8 (2.2%)	
Diabetes mellitus, n (%)	58 (11.2%)	23 (14.9%)	35 (9.6%)	0.079
Prior stroke or transient ischaemic attack, n (%)	40 (7.7%)	18 (11.7%)	22 (6.0%)	0.028
Coronary Artery Disease, n (%)	66 (12.7%)	27 (17.5%)	39 (10.7%)	0.033
Prior major bleeding	11 (2.1%)	8 (5.2%)	3 (0.8%)	0.004
Paroxysmal AF, n (%)	299 (57.7%)	76 (49.4%)	223 (61.3%)	0.012
Persistent or long-standing persistent AF, n (%)	219 (42.3%)	78 (50.6%)	141 (38.7%)	0.012
Concomitant therapy				
Amiodarone	86 (16.6%)	20 (13.0%)	66 (18.1%)	0.15
Dronedarone	11 (2.1%)	4 (2.6%)	7 (1.9%)	0.74
Flecainide	102 (19.7%)	21 (13.6%)	81 (22.3%)	0.024
Propafenone	14 (2.7%)	4 (2.6%)	10 (2.7%)	1.000
Sotalol	16 (3.1%)	4 (2.6%)	12 (3.3%)	0.787
ACE inhibitor or angiotensin receptor blocker	309 (59.7%)	102 (66.2%)	207 (56.9%)	0.047
Calcium channel blocker	122 (23.6%)	33 (21.4%)	89 (24.5%)	0.459
Diuretic	179 (34.6%)	62 (40.3%)	117 (32.1%)	0.076
Statin	186 (35.9%)	51 (33.1%)	135 (37.1%)	0.389
Beta blocker	364 (70.3%)	110 (71.4%)	254 (69.8%)	0.708
Digoxin	22 (4.3%)	10 (6.5%)	12 (3.3%)	0.099
Modified EHRA scale at baseline				0.096
mEHRA I, n (%)	35 (6.8%)	10 (6.5%)	25 (6.9%)	
mEHRA IIa, n (%)	129 (24.9%)	28 (18.2%)	101 (27.7%)	
mEHRA IIb, n (%)	164 (31.7%)	49 (31.8%)	115 (31.6%)	
mEHRA III, n (%)	180 (34.7%)	62 (40.3%)	118 (32.4%)	
mEHRA IV, n (%)	10 (1.9%)	5 (3.2%)	5 (1.4%)	
Rhythm at time of ablation				
Sinus rhythm, n (%)	358 (69.1%)	87 (56.5%)	271 (74.5%)	<0.001
Atrial fibrillation, n (%)	144 (27.86%)	63 (40.9%)	81 (22.3%)	
Atrial flutter, n (%)	9 (1.7%)	3 (1.9%)	6 (1.6%)	
Pacing, n (%)	7 (1.4%)	1 (0.6%)	6 (1.6%)	
Type of ablation				0.129
Pulmonary vein isolation	476 (91.9%)	147 (95.5%)	329 (90.4%)	
Pulmonary vein isolation with adjunctive ablation	39 (7.5%)	7 (4.5%)	32 (8.8%)	
Other	3 (0.6%)	0 (0%)	3 (0.8%)	
Ablation energy source				0.319
Radiofrequency, n (%)	323 (62.4%)	100 (64.9%)	223 (61.3%)	
Cryoablation, n (%)	154 (29.7%)	46 (29.9%)	108 (29.7%)	
Other	41 (7.9%)	8 (5.2%)	33 (9.1%)	

*Fisher exact test was used when cell size was small, that is, n<5; otherwise Pearson’s χ² was used for categorical and Wilcoxon rank-sum test was used when data is not normally distributed; otherwise two-sample t-test used for continuous.

AF, atrial fibrillation; AT, atrial tachycardia; BMI, body mass index; COPD, chronic obstructive pulmonary disease; mEHRA, modified European Heart Rhythm Association.

### Quality of life according to recurrent AT/AF

**Figure 1:**
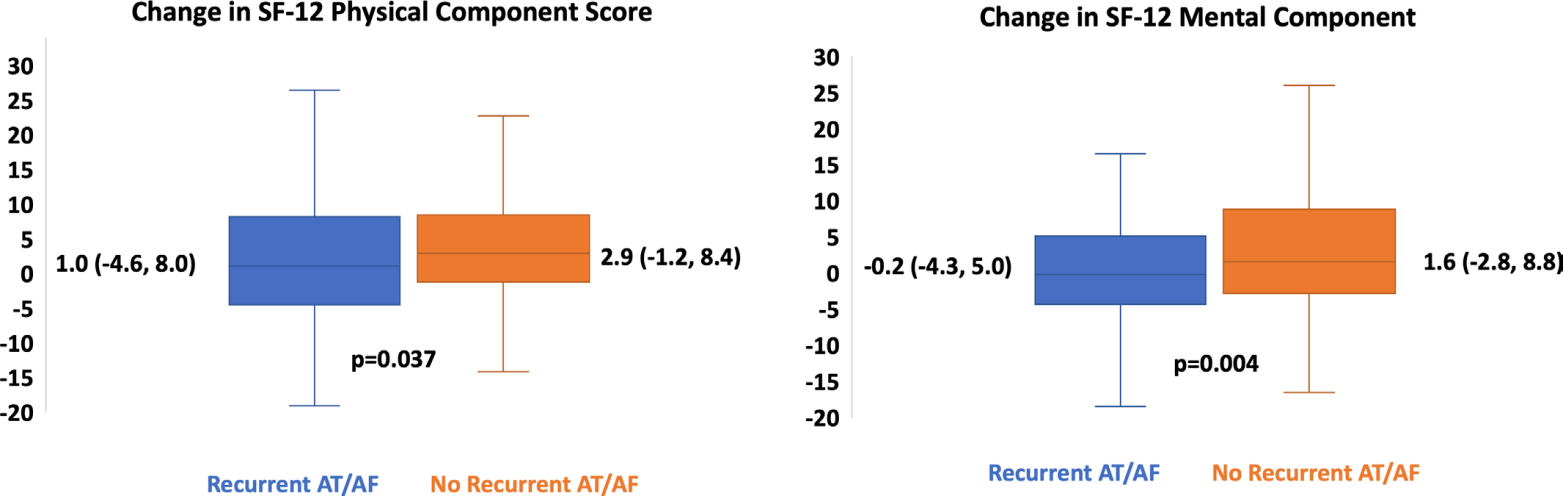
Change in the SF-12 physical and mental component scores according to the presence or absence of recurrent AT/AF. Shown in each box plot are the median changes with 25th and 75th percentiles in the SF-12 physical and mental component scores. The whiskers illustrate the maximum and minimum values. AF, atrial fibrillation; AT, atrial tachycardia.

**Figure 2:**
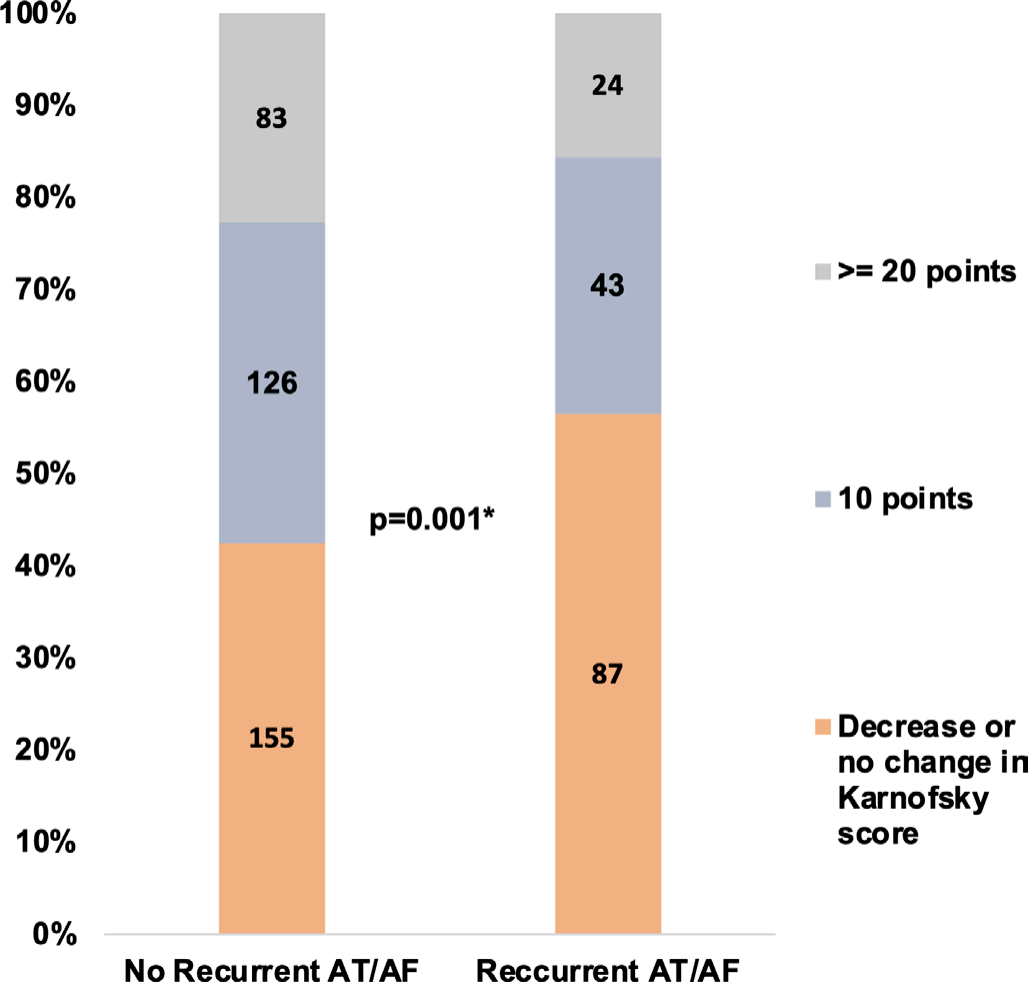
Shown in the bar graph are the changes in Karnofsky score categories according to the presence or absence of recurrent AT/AF. *P value is for the comparison in the change in median Karnofsky scores from baseline to follow-up. AF, atrial fibrillation; AT, atrial tachycardia.

At baseline (before ablation), patients with recurrent AT/AF had lower SF-12 physical component scores (42 (37, 49) vs 45 (39,52), p=0.004) and marginally higher MoCA scores (28 (26,29) vs 27 (25,29), p=0.002, table 2). After ablation, changes in QoL and cognitive function were better in those patients without recurrent AT/AF, including the median change in SF-12 physical component scores, SF-12 mental component scores, EQ-5D scores, and Karnofsky scores. Figure 1 illustrates the change in the SF-12 physical and mental component scores according to the presence or absence of recurrent AT/AF. Figure 2 illustrates the change in Karnofsky Index categories between those patients with and without recurrent AT/AF. Notably, in the overall cohort, the proportion with at least mild cognitive impairment (MoCA <26) declined from 30.3% (n=157) at baseline to 21.8% (n=113) at follow-up.

**Table 2:** Change in quality of life from baseline to end of study*

	All patients (n=518)	Recurrent AT/AF (n=154)	No recurrent AT/AF (n=364)	P value
SF-12 physical component				
Baseline, median (q1, q3)	45 (38 to 52)	42 (37 to 49)	45 (39 to 52)	0.004
End of study, median (q1, q3)	49 (42 to 54)	45 (38 to 52)	50 (44 to 55)	<0.001
Change, median (q1, q3)	3 (−2 to 8)	1 (−5 to 8)	3 (−1 to 8)	0.026
SF-12 mental component				
Baseline, median (q1, q3)	51 (43 to 58)	51 (45 to 58)	51 (43 to 58)	0.762
End of study, median (q1, q3)	54 (46 to 59)	52 (42 to 57)	55 (48 to 60)	0.003
Change, median (q1, q3)	1 (−3 to 8)	0 (−4 to 5)	2 (−3 to 9)	0.004
EQ-5D scores				
Baseline median (q1, q3)	0.8 (0.7 to 1.0)	0.8 (0.7 to 1.0)	0.8 (0.7 to 1.0)	0.457
End of study, median (q1, q3)	0.8 (0.7 to 1.0)	0.8 (0.7 to 1.0)	0.9 (0.7 to 1.0)	<0.001
Change, median (q1, q3)	0.0 (0.0 to 0.1)	0.0 (−0.1 to 0.1)	0.0 (0.0 to 0.2)	0.027
Karnofsky scale				
Baseline, median (q1, q3)	90 (80 to 90)	90 (80 to 100)	90 (80 to 90)	0.678
End of study, median (q1, q3)	100 (90 to 100)	90 (80 to 100)	100 (90 to 100)	<0.001
Change, median (q1, q3)	10 (0 to 10)	0 (0 to 10)	10 (0 to 10)	0.001
MoCA				
Baseline, median (q1, q3)	27 (25 to 29)	28 (26 to 29)	27 (25 to 29)	0.002
End of study, median (q1, q3)	28 (26 to 29)	28 (27 to 30)	28 (26 to 29)	0.003
Change, median (q1, q3)	1 (−1 to 2)	1 (−1 to 2)	1 (−1 to 2)	0.628
At least mild cognitive impairment (MoCA <26)				
Baseline, n (%)	157 (30.3%)	33 (21.4%)	124 (34.1%)	0.004
End of study, n(%)	113 (21.8%)	22 (14.3%)	91 (25%)	0.007

*Fisher exact test was used when cell size was small, that is, n<5; otherwise Pearson’s χ² was used for categorical and Wilcoxon rank-sum test was used when data is not normally distributed; otherwise two-sample t-test used for continuous.

AF, atrial fibrillation; AT, atrial tachycardia; MoCA, Montreal Cognitive Assessment.

ANCOVA demonstrated that the mean changes in QoL scores were greater among patients without recurrent AT/AF, when controlling for baseline measures as shown in table 3. The mean differences between those with and without recurrent AF, were not statistically significant at the p=0.05 level for any of the change in QoL measures except the Karnofsky score (4.9 (9.6) vs 7.9 (9.6), p<0.001). We also conducted a sensitivity analysis that assessed the proportion of patients with at least a 2.5 point increase in the PCS score. The proportion with a 2.5 point increase in the PCS was 44.8% (n=69/154) in those with recurrent AT/AF vs 52.2% (190/364) in those without recurrent AT/AF (p for difference after adjusting for baseline PCS=0.002). The proportion with a 2.5 point increase in the MCS was 35.7% (n=55/154) in those with recurrent AT/AF vs 46.7% (170/364) in those without recurrent AT/AF (p for difference after adjusting for baseline MCS=0.021).

**Table 3:** ANCOVA results comparing baseline and 3-month follow-up scores for QoL measures

	All patients (n=518)	Recurrent AT/AF (n=154)	No recurrent AT/AF (n=364)	P value
EQ-5D total				
Baseline	0.79 (0.22)	0.78 (0.21)	0.79 (0.22)	
End of study	0.83 (0.20)	0.78 (0.21)	0.84 (0.20)	
Change	0.04 (0.21)	0.00 (0.21)	0.05 (0.21)	0.131
SF-12 mental component				
Baseline	49.78 (9.61)	50.07 (9.22)	49.67 (9.78)	
End of study	51.79 (9.24)	49.94 (9.72)	52.58 (8.93)	
Change	2.01 (9.26)	−0.12 (9.09)	2.91 (9.19)	0.065
SF-12 physical component				
Baseline	44.31 (9.07)	42.57 (9.07)	45.04 (8.98)	
End of study	47.40 (8.69)	44.43 (9.33)	48.66 (8.09)	
Change	3.10 (8.24)	1.86 (8.93)	3.62 (7.88)	0.238
Karnofsky				
Baseline	86.3 (10.2)	85.8 (11.2)	86.5 (9.8)	
End of study	93.2 (9.7)	90.6 (11.4)	94.3 (8.6)	
Change	7.0 (9.7)	4.9 (9.6)	7.9 (9.6)	<0.001
MoCA				
Baseline	26.6 (2.8)	27.1 (2.5)	26.3 (2.9)	
End of study	27.2 (2.8)	27.7 (2.6)	27.0 (2.9)	
Change	0.6 (2.5)	0.6 (2.3)	0.7 (2.6)	0.924

The p value in this table corresponds to the effect of recurrent AT/AF group in an ANCOVA model with change from baseline as the dependent variable, and baseline values and recurrent AT/AF group as covariates.

AF, atrial fibrillation; ANCOVA, analysis of covariance; AT, atrial tachycardia; MoCA, Montreal Cognitive Assessment; QoL, quality of life.

### Associations between functional assessment and patient reported outcomes

In order to assess how improvements in functional status as measured by physician-determined mEHRA and Karnofsky functional status scores correlate with patient-reported outcomes (SF-12 and EQ-5D scores), we compared changes in these metrics. Table 4 details changes in QoL scores according to changes in Karnofsky scores. In patients with a decrease or no change in the Karnofsky score, the median change in the SF-12 physical component was 0.8 (−3.3, 6.5) compared with 3.3 (−0.4, 9.9) in those with a≥10 point Karnofsky improvement (p=0.002) and 5.4 (1.3, 11.2) in those with a≥20 point improvement in the Karnofsky score (p<0.001). Similarly, in patients with a decrease or no change in the Karnofsky score, the median change in the SF-12 mental component was 0.0 (−5.6, 5.8) compared with 1.6 (−1.9, 8.8) in those with a≥10 point Karnofsky improvement (p=0.035), and 2.2 (1.6, 10.0) in those with a≥20 point improvement in the Karnofsky score (p=0.013). Notably, the median change in the EQ-5D score was not materially different according to changes in the Karnofsky scores despite small but statistically significant improvements. The changes in patient-reported outcomes according to changes in mEHRA scores are shown in table 5. Patients with >1 class improvement in the mEHRA had greater increase in median SF-12 physical component scores at the end of the study compared with those with decreased or no change in mEHRA classification (p=0.033).

**Table 4:** Changes in quality of life according to changes in Karnofsky score*

	Decrease or no change in Karnofsky score (n=242)	≥10 point improvement in Karnofsky score (n=276)	≥20 point improvement in Karnofsky score (n=107)
Change in SF-12 physical component score compared with baseline (Δ PCS)			
Mean (SD)	1.5 (8.2)	4.5 (8.0)	6.5 (7.9)
Median (25th, 75th)	0.8 (−3.3 to 6.5)	3.3 (−0.4 to 9.9)	5.4 (1.3 to 11.2)
Min, Max	−19 to 30	−17 to 26	−16 to 26
P value (compared with decrease or no change)		0.002	<0.001
Change in SF-12 mental component score compared with baseline (Δ MCS)			
Mean (SD)	0.3 (9.4)	3.5 (8.9)	4.4 (9.3)
Median (25th, 75th)	0.0 (−5.6 to 5.8)	1.6 (−1.9 to 8.8)	2.2 (−1.6 to 10.0)
Min, Max	−29 to 26	−22 to 44	−21 to 44
P value (compared with decrease or no change)		0.035	0.013
Change in EQ-5D score compared with baseline (Δ EQ-5D)			
Mean (SD)	0.0 (0.2)	0.1 (0.2)	0.1 (0.2)
Median (25th, 75th)	0.0 (−0.1 to 0.1)	0.0 (0.0 to 0.2)	0.0 (0.0 to 0.2)
Min, Max	−1 to 1	−1 to 1	−1 to 1
P value (compared with decrease or no change)		0.040	0.001

The patients with ≥20 point improvement in the Karnofsky score (n=107) are also included in the group of patients with ≥10 point improvement (n=267). Unadjusted p values are from median comparison with ‘Decrease or No Change in Karnofsky Score’ group.

**Table 5:** Changes in quality of life according to changes in mEHRA*

	Decrease or no change in mEHRA (n=81)	Any improvement in mEHRA (n=437)	1 Class improvement in mEHRA (n=146)	>1 Class Improvement in mEHRA (n=291)
Change in SF-12 physical component score compared with baseline (Δ PCS)				
Mean (SD)	0.6 (8.3)	3.6 (8.2)	1.7 (7.7)	4.5 (8.2)
Median (25th, 75th)	0.9 (−5.8 to 5.8)	2.7 (−1.4 to 8.8)	0.6 (−2.2 to 5.5)	3.8 (−1.0 to 9.6)
Min, Max	−19 to 26	−18 to 18	−19 to 26	−18 to 30
P value (compared with decrease or no change)		0.277	0.851	0.033
Change in SF-12 mental component score compared with baseline (Δ MCS)				
Mean (SD)	0.9 (8.9)	2.2 (9.3)	1.7 (8.0)	2.5 (9.9)
Median (25th, 75th)	0.0 (−3.3 to 5.0)	1.4 (−3.1 to 8.3)	1.6 (−2.4 to 7.3)	1.2 (−3.2 to 8.9)
Min, Max	−29 to 27	−22 to 44	−22 to 22	−24 to 44
P value (compared with decrease or no change)		0.116	0.08	0.259
Change in EQ-5D score compared with baseline (Δ EQ-5D)				
Mean (SD)	0.0 (0.2)	0.0 (0.2)	0.0 (0.2)	0.0 (0.2)
Median (25th, 75th)	0.0 (−0.1 to 0.1)	0.0 (0.0 to 0.1)	0.0 (0.0 to 0.1)	0.0 (0.0 to 0.2)
Min, Max	−1 to 1	−1 to 1	0 to 1	−1 to 1
P value (compared with decrease or no change)		0.343	0.879	0.179

*Unadjusted p values from median comparisons with 'Decrease or No Change in mEHRA' group.

mEHRA, modified European Heart Rhythm Association.

## Discussion

Catheter ablation is performed to reduce arrhythmia burden, minimise symptoms and to improve QoL. While there are several trials that examined the impact of catheter ablation on QoL compared with medical therapy, including the recent CABANA trial,^3^ few have examined the impact of recurrent AT/AF after ablation on changes in QoL, functional status and patient reported outcomes. In our analysis of recurrent AT/AF after ablation in the AXAFA trial, we found that the raw changes in QoL (SF-12 physical and mental scores), EQ-5D and functional (Karnofsky status scores) improved more in patients without recurrent AF. After adjustment using ANCOVA, similar trends were observed although statistically significant improvement was greater only as measured with the Karnofsky score. Finally, we found that cognitive function as assessed by MoCA scores improved slightly after ablation regardless of recurrent AF.

Catheter ablation is an established therapy for patients with medically refractory symptomatic AF. While catheter ablation has led to improved cardiovascular outcomes in patients with heart failure and left ventricular dysfunction, the primary indication for ablation is to reduce symptoms and improve QoL. The aggregate evidence available from clinical trials demonstrates improvement in QoL after ablation. In the ThermoCool AF trial, patients who underwent ablation experienced significant improvement in SF-36 scores (mean+6.9 points for mental, +6.6 for physical) at 9 months of follow-up.^2^ Similarly, in the Catheter Ablation compared with optimised Pharmacological Therapy for Atrial Fibrillation (CAPTAF) trial, in which QoL was the primary endpoint, catheter ablation led to superior QoL compared with medical therapy at 12 months of follow-up.^4^ Most recently, the CABANA trial demonstrated that catheter ablation led to significant improvements in QoL that were maintained at 5 years after ablation when compared with medical therapy.^3^ Despite randomised trials comparing QoL with ablation versus medical therapy, few data are available comparing QoL, functional status and patient-reported outcomes in patients according to recurrence of atrial arrhythmias after ablation.

Consistent with our hypothesis, we observed that improvement in functional status was greater in those patients without recurrent AT/AF, as reflected by highly significant improvement in Karnofsky scores. However, we did not identify greater improvements in QoL in those without recurrent AT/AF. While the median changes in the SF-12 physical and mental scores were more favourable in patients without recurrent AT/AF, ANCOVA did not identify statistically significant greater improvement SF-12 physical (p=0.238) or mental scores (p=0.065) in those without recurrent AT/AF compared with patients with recurrent AT/AF. Finally, while the change in MoCA scores was not different between those with and without recurrent AT/AF after the 3-month follow-up, the proportion of the overall cohort with mild or greater cognitive impairment decreased from 30% to 22%. This cognitive improvement could also be related to an increased familiarity of the test by patients. Nonetheless, this finding is reassuring, particularly in light of findings of asymptomatic cerebral emboli in patients undergoing catheter ablation.^6 7^

Patient-reported outcomes are defined as any report of the status of a patient’s health that comes directly from the patient, without interpretation of the patient’s response by a clinician or anyone else. Patient-reported outcomes have been increasingly emphasised by regulatory bodies, including the Food and Drug Administration. AXAFA included several patient-reported outcomes including the physical and mental components of the SF-12 and EQ-5D. There are few data regarding how clinician-derived and patient-reported assessments of improvement in functional status and QoL relate to one another. Results from the ORBIT registry suggest that changes in patient-reported QoL with the AFEQT score do correlate with the EHRA scores.^17^ In AXAFA, we found that improvements to Karnofsky score appeared to be reflected by improvements in patient-reported outcomes including the SF-12 physical and mental components.

### Limitations

There are several limitations that should be kept in mind when considering these data. This is a secondary analysis of data from a randomised controlled trial and QoL data were not available for all patients. While the hypothesis tested in our analysis was whether changes in QoL after ablation differ according to the presence or absence of recurrent AT/AF, we did not have a comparator group who did not undergo ablation. Additionally, the changes in QoL were analysed at 3 months and therefore reflect intermediate term outcomes after AF ablation. Furthermore, despite of using different test versions, a learning effect by serial testing has to be taken into account.

## Conclusion

AF ablation resulted in very good functional status in this large cohort of patients with stroke risk factors. Patients without recurrent AT/AF experience greater improvement in functional status after AF ablation. Future studies should examine how changes in QoL, cognition and functional status vary according to arrhythmia recurrence in long-term follow-up.

**Key messages**

**What is already known on this subject?**

- Catheter ablation of atrial fibrillation (AF) improves quality of life.

**What might this study add?**

- This study examines how quality of life changes according to outcomes after AF ablation.

**How might this impact on clinical practice?**

- Patients without recurrent atrial tachycardia/AF experience greater improvement in functional status after AF ablation. Assessing patient-reported outcomes after ablation is important to help guide treatment and improve outcomes.
